# Non-Pharmacological Strategies and Interventions for Effective COVID-19 Control: A Narrative Review

**DOI:** 10.3390/jcm12206465

**Published:** 2023-10-11

**Authors:** Ludwig Serge Aho Glele, Alexis de Rougemont

**Affiliations:** 1 Epidemiology and Infection Control Department, University Hospital of Dijon, 21000 Dijon, France; 2 National Reference Centre for Gastroenteritis Viruses, Laboratory of Virology, University Hospital of Dijon, 21000 Dijon, France; alexis.de-rougemont@u-bourgogne.fr

**Keywords:** COVID-19, SARS-CoV-2, prevention, non-pharmacological interventions, standard precautions, airborne and droplet transmission, personal protective equipment, mask, ventilation, room occupancy, testing for SARS-CoV-2

## Abstract

The COVID-19 pandemic had a devastating impact on the world, causing widespread illness and death. Focusing on prevention strategies to limit the spread of the disease remains essential. Despite the advent of vaccines, maintaining a vigilant approach to prevention remains paramount. We reviewed effective strategies to prevent COVID-19 transmission, including various prevention measures and interventions and both established practices and unresolved issues that have been addressed in meta-analyses, literature reviews, or in the health care context. Standard precautions are the cornerstone of infection control, with hand hygiene and mask use as key components. The use of surgical masks is recommended to prevent droplet transmission, while eye protection is recommended in combination with masks. In terms of room occupancy, ventilation is critical in reducing the risk of transmission in poorly ventilated environments. Chemical disinfection of indoor air with Triethylene glycol-based products can provide safe additional protection. Since viral RNA detection on surfaces does not necessarily indicate infectivity, the risk of transmission by surface contact remains low if surfaces are properly maintained and hand hygiene is practiced regularly. Thus, prevention of SARS-CoV-2 transmission requires a multifaceted approach, including reducing particle emissions from infected persons by wearing masks, eliminating aerosols by ventilation and air treatment, ensuring physical separation, and protecting exposed persons with masks and eye protection.

## 1. Introduction

The COVID-19 (Coronavirus Disease 2019) pandemic has had an immense global health, social, and economic impact. As the world grapples with the challenges posed by the SARS-CoV-2 virus (Severe Acute Respiratory Syndrome Coronavirus 2), it is critical to focus on prevention strategies to limit the spread of the disease, even in the presence of vaccines.

The end of the public health emergency associated with COVID-19 in many countries could lead one to believe that COVID-19 is no longer relevant.

COVID-19 is still relevant, as evidenced by data on SARS-CoV-2 circulation and the emergence of new variants such as XBB.1.5 [1,2]. Current data on COVID-19 incidence in Europe (7 September 2023) show that signals of SARS-CoV-2 transmission have increased in recent weeks from previously very low levels [3].

COVID-19 also remains relevant because of its impact on vital prognosis [4], with hospital mortality currently higher than that of influenza. Certainly, hospital mortality from COVID-19 has declined over time. This decline is likely to be related to several factors, such as increased immunity (vaccination and prior infection) and improved clinical care. However, over the same period, hospital mortality rates for influenza decreased slightly but remained lower than those for COVID-19 (3.7% vs. 6%) [5].

In addition, long-term COVID or post-COVID syndrome may continue to affect a very high proportion of patients who have had the disease [6,7].

Prevention of COVID-19 is based on pharmacological measures, such as vaccination, and non-pharmacological measures, such as wearing a mask.

Vaccination (including boosters) helps to prevent SARS-CoV-2 infection, illness, hospitalization, and death. This overall effectiveness varies according to the assessment criteria mentioned above, and the duration of protection also varies. For example, the effectiveness of a fourth dose of conventional mRNA vaccines (BT162b2 or mRNA-1273) is low and short-lived in preventing infection with SARS-CoV-2 in its predominant variant (Omicron) [8]. However, the efficacy of these conventional mRNA vaccines against severe symptomatic infection, hospitalization, and death is high. New bivalent and active vaccines against omicron variants are now available [9].

Both vaccination and infection have led to an increase in population immunity. This could suggest that non-pharmacological interventions are of less benefit in the prevention of COVID-19. However, interventions such as wearing a mask or adequate ventilation of premises remain effective and recommended strategies for the prevention of respiratory infections [10].

The aim of this narrative review (NR) [11] is to present and summarize effective prevention strategies for COVID-19, focusing primarily on meta-analyses (MA) and literature reviews (LR). In addition, interventions that have generated debate within the scientific community will be discussed.

Effective prevention measures not only protect individuals from infection but also contribute to the collective effort to reduce transmission within communities and the burden on healthcare systems. In general, prevention can be linked to the natural history of the disease. The classification of the natural history of a disease can be divided into five stages: underlying, susceptible, subclinical, clinical, and recovery/disability/death. The corresponding preventive health measures can be grouped into three categories: primordial prevention, primary prevention, secondary prevention, and tertiary prevention [12].

Primordial prevention aims to address the underlying social and environmental conditions that promote the onset of disease. In the case of COVID-19, examples of primordial prevention may include promoting health education campaigns that focus on hygiene practices, such as hand washing and respiratory etiquette, to prevent the spread of the virus. The primary prevention aims to prevent the disease from occurring in healthy individuals through promoting measures including vaccination campaigns to provide immunity to SARS-CoV-2, thereby reducing the risk of infection and severe illness, and transmission prevention strategies such as mask wearing, physical distancing, good hand hygiene practices, and antiviral air treatment (e.g., UV light, air filtration, and chemical disinfection such as Triethylene Glycol (TEG)). Secondary prevention focuses on early detection and intervention in people with subclinical forms of COVID-19 by implementing regular testing and screening programs in high-risk settings to identify asymptomatic or pre-symptomatic individuals and contact tracing efforts to identify and isolate individuals previously in close contact with confirmed cases. Tertiary prevention aims to reduce the severity of the disease and associated complications in infected people by providing medical care and treatments (e.g., antiviral therapies and oxygen therapy), supportive care to individuals with severe disease to improve outcomes and minimize complications, or rehabilitation programs to support the recovery of individuals who have experienced long-term effects or disabilities due to COVID-19. At last, quaternary prevention aims to protect individuals from unnecessary or potentially harmful medical interventions, such as avoiding the unnecessary use of certain medications or treatments that have not been shown to be effective in the treatment of COVID-19.

Here, we review a comprehensive range of preventive measures and strategies used to control COVID-19, including the scientific evidence supporting these interventions, and highlight their role in reducing the risk of transmission. We have chosen to focus on the prevention of COVID-19 in hospital settings in a manner that is relatively consistent with the practices of health care facilities. Consequently, certain types of prevention, such as primordial prevention, will not be discussed in this article. We also address the importance of non-pharmaceutical interventions in preventing the spread of SARS-CoV-2, including standard precautions and other precautions, personal protective equipment (PPE), physical distancing, air treatment, and proper ventilation in enclosed spaces. Alongside non-pharmaceutical interventions, we also emphasize the fundamental role of vaccination in preventing severe illness, hospitalization, and death, consider the importance of testing as a preventive measure, and examine the use of diagnostic tests to identify and isolate infected individuals, with a particular focus on asymptomatic cases. Finally, we will show that a multifaceted approach to prevention is essential.

## 2. Materials and Methods

We conducted a literature search using electronic scientific resources such as PubMed, Science Direct, Google Scholar, and MedRxiv between 2020 and August 2023. Our aim was to identify relevant English-language articles using terms such as prevention, “standard precaution”, “contact precaution”, droplet, airborne, “surgical mask”, “N95 mask”, “FFP2 mask”, ventilation, surface, “non-pharmacological” (or “non-pharmaceutical”), “preventive measure” (or “preventive intervention”), etc., in relation to “COVID-19”, “SARS-CoV-2”, or “severe acute respiratory syndrome”. 

In addition to LR and MA, we also looked for relevant original articles on the topic.

To supplement our search, we manually identified and included references to recent research, and we also thoroughly checked the reference lists of selected literature.

The following eligibility criteria were used to select articles for inclusion in this review: Articles were published in English; they were either reviews (narrative or MA) or original research articles.

## 3. Results

### 3.1. Precautions

Standard precautions (SP) [13] prevent the spread of infections in healthcare settings. SP has been adopted worldwide and is regularly updated. Hand hygiene is a key component of SP, with the use of hydroalcoholic solutions in hospitals. Hospitals have been under pressure to produce their own products, some of which are based on WHO (World Health Organization) formulations (the original and modified). Published tests have shown that these formulations are active against SARS-CoV-2 [14].

Uncertainties about the relative importance of droplet and aerosol transmission mean that there may be discrepancies in recommendations between different agencies. For example, for MERS-CoV (Middle East Respiratory Syndrome-related Coronavirus; formerly 2012-nCoV or nCoV), the WHO has historically advocated “contact + droplet” precautions versus “contact + air” precautions for the CDC (Centers for Disease Control and Prevention) [15].

See the Personal Protective Equipment section for details.

### 3.2. Personal Protective Equipment (PPE)

#### 3.2.1. Masks

Preamble: See the following FDA (US Food and Drug Administration) page [16] for details of the different types of masks. The FFP2 mask is the European equivalent of the N95 mask (USA) or the KN95 (China).

In order to prevent droplet transmission, wearing a surgical mask is recommended [17]. This recommendation is based on abundant literature related to respiratory viruses, including influenza, and several Randomized Controlled Trial (RCTs) on masks alone [18,19] or associated with hand hygiene [20]. RCTs show no significant difference or inferiority in terms of reduction in the incidence of infection between the surgical mask and the FFP2/N95 mask [18,19]. In fact, for coronavirus, low-level evidence suggests that surgical masks and FFP2/N95 masks provide similar protection for Health Care Workers (HCW) during the Aerosol Generating Procedure (AGP) [21]. Kunstler et al. [22] performed a MA to compare N95/FFP2 vs. surgical masks in order to prevent SARS-CoV-2 infection. Twenty-one studies were included, with most having a high risk of bias. OR between N95/FFP2 and surgical mask was 0.85 (95% CI: 0.72–1.01). HCW experienced significantly more adverse events (headaches, respiratory distress, facial irritation, and pressure-related injuries) when wearing N95/FFP2 compared to a surgical mask. For the prevention of COVID-19, whether in community settings or in health care settings, recently published observational studies provided insufficient evidence for N95/FFP2 vs. surgical masks [23].

In late 2022, Loeb et al. [24] published a RCT comparing the effectiveness of medical masks versus N95 respirators in preventing COVID-19 among 1009 HCW providing routine care. The trial took place from May 2020 to March 2022 in 29 healthcare facilities in Canada, Israel, Pakistan, and Egypt. The primary outcome measure was confirmed to be COVID-19 by reverse transcriptase polymerase chain reaction (RT-PCR). The results showed that, in the intention-to-treat analysis, the incidence of RT-PCR-confirmed COVID-19 was similar in the medical mask group (10.46%) compared with the N95 respirator group (9.27%) (Hazard Ratio [HR], 1.14 [95% CI: 0.77–1.69]). However, an unplanned subgroup analysis by country showed variability in the results. There were adverse events in both groups. There were 47 (10.8%) intervention-related adverse events in the medical mask group and 59 (13.6%) in the N95 respirator group. This study concludes that among HCW providing routine care to patients with COVID-19, the overall estimates exclude a doubling of hazard with RT-PCR-confirmed COVID-19 for medical masks compared with the HRs of RT-PCR-confirmed COVID-19 for N95 respirators. Limitations include potential acquisition of SARS-CoV-2 through household and community exposure, heterogeneity between countries, uncertainty in effect estimates, differences in self-reported adherence, differences in baseline antibodies, and between-country differences in circulating variants and vaccination. Early in the pandemic, mask use in Asian countries showed significant protection against COVID-19 [25].

Chou et al. [23] recently published a MA to update the understanding of the effectiveness of masks in preventing SARS-CoV-2 infection in both community and healthcare settings. They analyzed RCT and observational studies of N95 respirators, surgical masks, and cloth masks. The results showed that mask use in community settings may be associated with a small reduction in the risk of SARS-CoV-2 infection, based on the results of two RCTs and seven observational studies. In routine patient care settings, surgical masks and N95 respirators were similarly effective in preventing SARS-CoV-2 infection, according to one new RCT and four observational studies. However, limitations in the available evidence prevented a comprehensive evaluation of other mask types and comparisons. Finally, although Chou et al. [23] suggest that masks may provide only a modest reduction in the risk of SARS-CoV-2 infection in community settings, other evidence indicates more robust protection from masks. The effectiveness of N95 respirators in hospital settings cannot be definitively determined.

#### 3.2.2. Eye Protection

There is evidence that SARS-CoV-2 can either infect ocular surface cells directly or be transported by tears through the nasolacrimal duct to infect the nasal or gastrointestinal epithelium [26,27]. Goggles and visors, which are anti-projection devices and therefore have no filtering capacity, should be combined with mask use [28]. According to the nationwide matched case-control study by Belan et al. [29], eye protection (OR: 0.57; 95% CI: 0.37–0.87) and also wearing a gown (0.58; 0.34–0.97) were protective when caring for COVID-19 patients. Therefore, the implementation of eye protection measures is proving to be an effective strategy in reducing the transmission of COVID-19, particularly in the care of patients affected by the virus.

The above data on the effectiveness of PPE (e.g., masks and eye protection) in preventing COVID-19 and the results of additional studies are summarized in Table 1.

### 3.3. Facility Management Strategies

#### 3.3.1. Ventilation and Negative Pressure

The risk of transmission of the virus from an asymptomatic carrier can vary depending on several parameters: distance, room ventilation, and case activity. Jones et al. [37] propose a risk assessment table that takes these parameters into account. The authors state that high-density environments are most at risk when HCW occupies poorly ventilated spaces: rest rooms, meeting rooms, or changing rooms. However, “high density” implies the presence of a large number of people in a small space. It can also refer to the presence of “superspreaders” in a volume that appears ad hoc given the number of people [37].

Early on, negative pressure isolation rooms (NPIR) have been proposed for COVID-19 patients [38]. They are designed to maintain a lower air pressure than the surrounding area, preventing contaminated air from escaping and potentially infecting others. When an infected person is placed in a NPIR, air is continuously drawn into the room and then filtered and exhausted to the outside. This helps to minimize the concentration of viral particles in the air, reducing the risk of transmission to healthcare workers and other patients in the facility. It is worthy to note that NPIR alone is not a foolproof solution and should be used in conjunction with other preventive measures such as PPE and appropriate hygiene practices. In addition, the effectiveness of NPIR in preventing COVID-19 transmission depends on several factors, including proper design, construction, and adherence to established guidelines and protocols. Overall, NPIRs are an essential tool in the management of COVID-19 cases by reducing the risk of airborne transmission and protecting healthcare workers and others from exposure to the virus. 

International guidelines and recommendations for ventilation in modified NPIRs for COVID-19 patients were compared by Chung-Yen Chen et al. [39]. Modified “quasi-negative pressure” isolation wards were found to be a feasible, inexpensive, safe, and effective measure to control nosocomial outbreaks. However, preventive measures such as CO2 monitoring, mechanical ventilation, and air purification should also be carefully considered [40], as well as the chemical treatment of the air with a TEG-based product that has been shown to disinfect a SARS-CoV-2 surrogate at concentrations that are harmless to humans [41].

Thus, the minimum ventilation volume required for an isolation unit should be determined based on the severity of COVID-19 patients. Mechanical ventilation remains the mainstay for achieving this requirement, although support from recirculation may also be helpful. In addition to adequate tidal volumes, ‘clean to less clean’ directional airflow remains the golden rule for indoor ventilation solutions. This means that air should flow from clean areas to less clean areas, such as from the corridor to the isolation unit. Virus-laden exhaust air should be treated with HEPA/UV (High-Efficiency Particulate Air Filter/Ultraviolet) equipment, where HEPA filters can capture particles as small as 0.3 microns, while UV light and TEG can kill viruses and bacteria.

In essence, infection control strategies involving room ventilation are highly dependent on the specific characteristics and use of each room. In order to implement effective infection control measures, a thorough analysis of individual rooms is essential. The selection of the most appropriate and feasible ventilation system should be based on a number of factors, including available vents, room height, volume, use, economic and energy requirements, and structural considerations [42].

#### 3.3.2. Room Management and Occupancy

Room management is an important part of organizing COVID-19 patient care in healthcare facilities. Bertuzzi et al. [43] conducted a systematic review (SR) to assess the impact of single rooms versus shared accommodation on inpatient outcomes not specific to COVID-19 (clinical, humanistic, and economic outcomes). While the overall benefit of single rooms was inconclusive, they did show a small clinical advantage for critically ill patients, particularly neonates in intensive care. 

However, given that hospitalization in a double room compared to a single room increases the risk of nosocomial infections such as influenza [44], we can assume that the same is true for COVID-19. Karan et al. [45] assessed SARS-CoV-2 transmission between patients in shared rooms in an academic hospital during the first wave. Out of 11,290 patients admitted to shared rooms, 25 tested positive. Of 31 exposed roommates, 12 (39%) tested positive within 14 days. Transmission was associated with positive PCR at Ct ≤ 21 (the cycle threshold). However, Hyun et al. [46], observed no statistically significant differences between two randomized groups, including COVID-19-positive patients cared for in shared or single rooms. The results suggested that shared accommodation for patients with mild symptoms could be an alternative to single-room occupancy during the COVID-19 pandemic. Trannel et al. [47] assessed the incidence following exposure in shared patient rooms in a tertiary care center with 38,142 patient days in shared rooms between July 2020 and May 2021. The incidence of SARS-CoV-2 exposure in a shared room was 1.8 per 1000 patient days in a shared room. A total of 37 (52.9%) exposed roommates completed follow-up testing, of which 8 (21.6%) converted. This secondary attack rate was comparable to that reported in household exposures. The median time to conversion was 5 days (range: 2–11). The incidence of conversion after exposure was 0.4 per 1000 patient days in a shared room. None of the conversions were related to an AGP. There were no statistically significant differences in age, sex, or the presence of infectious patient symptoms (source) between those who converted and those who did not. Williams et al. [48] conducted a study to assess the effectiveness of universal admission testing for SARS-CoV-2 on 28,603 patients in preventing transmission in shared patient rooms at a large academic medical center. Universal admission testing was effective in preventing transmission, especially since the emergence of the SARS-CoV-2 omicron variant. The yield of identifying infectious asymptomatic cases more than doubled during the Omicron era. Of the asymptomatic patients identified as infectious, a third were temporarily admitted to a shared room. Among contacts of asymptomatic patients, 4.1% developed a SARS-CoV-2 infection. The authors then concluded that universal admission testing is an effective way to prevent transmission of SARS-CoV-2 in shared patient rooms. In fact, the implementation of testing alongside other infection prevention and control (IPC) measures has facilitated the safe provision of patient care in a hospital where shared patient rooms predominate [49].

In any case, hospitalization in a double room does not impede the provision of appropriate care, even in the context of COVID-19. Therefore, hospitals should implement measures to reduce exposure in shared rooms.

### 3.4. Virus Detection and Surface Disinfection

#### 3.4.1. Surface Role

The role of surfaces in SARS-CoV-2 contamination has been debated since the beginning of the COVID-19 pandemic. If the virus survives on surfaces, what are the conditions for its survival? Can surface transmission be quantified? Can surface disinfection reduce the risk of SARS-CoV-2 infection?

Several articles have highlighted the presence of SARS-CoV-2 viral RNA on surfaces and in the air. One of the best known is that of Van Doremalen et al. [50]; however, the experimental conditions did not reflect reality: transposition to emissions produced by an individual (e.g., spontaneous breathing in a room); vast excess of virus tested on surfaces compared to actual viral loads found in real life [51]. However, the survival in the environment and the transmission to a susceptible host depend on many factors specific to the virus, the host, and the environmental conditions (temperature, humidity, etc.) [51,52,53], while even the presence of viral RNA does not necessarily imply infectiousness [54,55].

The half-life of SARS-CoV-1 [56] decreases with lower input concentrations of virus. However, the stability of SARS-CoV-2 on various inanimate surfaces and its potential for surface transmission remain a concern. Xu et al. [57] studied the effect of variations in temperature, relative humidity (RH), and initial virus titer and the factors influencing the half-life of SARS-CoV-2 on virus stability on six different contact materials: plastic, metal, glass, protective equipment, paper, and fabric. The half-lives of different contact materials ranged from 2 to 10 h, up to 5 days, and as low as 30 min at a temperature of 22 °C. In contrast, on porous surfaces, the half-life was typically 1 to 5 h, up to 2 days, and as short as 13 min at 22 °C. On non-porous surfaces, the half-life was longer, generally 5 to 9 h, up to 3 days, and as low as 4 min at the same temperature. Thus, the half-life of SARS-CoV-2 on non-porous surfaces was longer than that on porous surfaces. In addition, the half-life of the virus decreased with increasing temperature, while RH had a stable negative inhibitory effect only within a certain humidity range. Therefore, even if viral RNAs can last several hours or days on surfaces, the viral load decreases rapidly. The risk of transmission by contact therefore appears to be low and easily manageable with regular surface maintenance and frequent hand hygiene.

If surfaces play a role in the transmission of COVID-19, can we assess the role of contaminated surfaces through studies evaluating the effectiveness of surface disinfection in preventing the occurrence of COVID-19? Unfortunately, no RCTs evaluating the effectiveness of surface disinfection in preventing the occurrence of COVID-19 have been published yet. A review of randomized controlled trials (RCTs) by Thomas et al. [58] found that only 5 of the 14 RCTs reviewed showed a reduction in the risk of infection; however, the authors focused only on multidrug-resistant organisms (MDROs) and Clostridioides difficile; therefore, the findings may not be relevant to COVID-19. Ray et al. [59] also focused on C. difficile and found that an environmental disinfection intervention improved the thoroughness and effectiveness of cleaning but did not reduce the incidence of healthcare-associated C. difficile infections. Therefore, the evidence for the effectiveness of surface disinfection in preventing the occurrence of COVID-19 is limited. The US CDC has, however, estimated the chance of infection from a surface to be less than 1 in 10,000 [60]. As a result, we can conclude that surfaces may play a very minor role in the transmission of COVID-19, although the evidence is limited.

#### 3.4.2. SARS-CoV-2 Testing in Asymptomatic Patients

Indications for direct testing for SARS-CoV-2 in asymptomatic individuals in health care settings, with the aim of preventing SARS-CoV-2 transmission in these settings, have been formulated in the European Society of Clinical Microbiology and Infectious Diseases (ESCMID)-approved guidelines developed using the GRADE (Grading of Recommendations, Assessment, Development, and Evaluations) approach [61]. As an example, “the Panel recommends preoperative testing of asymptomatic patients 48–72 h before elective surgery requiring anesthesia to reduce the exposure of HCWs in settings with a high transmission rate and/or low vaccination coverage and/or limited access to PPE” (conditional recommendation, QoE: very low).

The benefits of “asymptomatic screening” are unclear when added to other infection prevention measures. In line with ESCMIDs position, the Society for Healthcare Epidemiology of America (SHEA) advises against the routine, universal use of asymptomatic screening in healthcare settings [38,62]. SHEA states that “admission screening may be beneficial during times of increased virus transmission in some settings where other layers of controls are limited (e.g., behavioral health, congregate care, or shared patient rooms); however, widespread routine use of admission asymptomatic screening is not recommended over strengthening other infection prevention controls”.

In their Viewpoint article, Brust et al. [63] state that no increase in hospital-acquired COVID-19 has been documented since the discontinuation of admission testing. This statement is based on an analysis of the few published experiences with cessation of testing for SARS-CoV-2 in asymptomatic patients. Most of the studies were retrospective observational analyses. Brust et al. [63] could not find any prospective evaluations involving control units or hospitals.

Thus, evidence on the effectiveness of direct testing for SARS-CoV-2 in asymptomatic persons in healthcare settings is limited. ESCMIDs guidelines recommend pre-operative testing of asymptomatic patients in some settings; however, the benefit of this testing is unclear. SHEA advises against the routine, universal use of asymptomatic screening in healthcare settings.

## 4. Discussion and Conclusions

We conducted this LR article to report effective prevention strategies for COVID-19, focusing primarily on MA and LR articles. Interventions that have generated debate were also discussed.

According to several international guidelines [10,64,65], the prevention of transmission of SARS-CoV-2, whatever the variant, is based on a series of essential complementary and simultaneous measures. Overall, the evidence from the literature suggests that non-pharmacological preventive measures are effective in preventing the spread of COVID-19. These measures should be used in combination with vaccination to protect individuals from the virus, reduce virus emissions, protect from the projection/aerosolization of biological products from infected people (e.g., masks, goggles, visors, or shields), eliminate aerosols through the disinfection of surfaces and the ventilation/aeration of premises, or respect a physical distance of 2 m. However, uncertainties about the relative importance of droplet and aerosol transmission mean that there may be discrepancies in recommendations between different agencies [15].

Several LR or MA articles have addressed the prevention of COVID-19. They concern global public health measures such as quarantine, social distancing, etc. [66,67,68], or are specific to one type of measure, such as mask use. For example, the MA by Chu et al. [69], published at the beginning of the pandemic, addresses mask and eye protection as a means to prevent the occurrence of COVID-19. Chou et al. [23] conducted an updated MA study of the effectiveness of mask wearing in preventing SARS-CoV-2, while Alkhalaf et al. [70] focused on dentistry, and Utzet et al. [71] focused specifically on HCWs.

In this latest study, the authors evaluated the effectiveness of non-pharmacological preventive measures for COVID-19 in HCWs before the vaccination era on a dynamic cohort of 5543 HCWs employed for at least one week in a Spanish hospital in 2020. Negative binomial regression models were used to assess the incidence rate and rate ratio (RR) during two different waves (15 March to 21 June and 22 June to 31 December), adjusting for natural immunity from the first wave and contextual factors. There was a significant reduction in the average COVID-19 incidence rate per 1000 working days from 0.82 (CI 95%: 0.73–0.91) to 0.39 (0.35–0.44). After adjustment for natural immunity and contextual factors, the adjusted RR was 0.54 (0.48–0.87). The authors concluded that the remarkable decrease in COVID-19 incidence was largely due to improvements in non-pharmacological preventive measures as a whole, which calls for a more specific and individualized evaluation of the effectiveness of the selected non-pharmacological preventive measures.

Our narrative review focused on MA. However, the reported results of some MAs conducted to assess the effectiveness of preventive interventions should be considered with caution. Some reasons for this are the fragility of the results [72], the MA of observational studies (instead of randomized trials), the inclusion of studies with a low level of evidence, and the use of suboptimal statistical methods to account for studies reporting few or no events. For example, the MA by Kunstler et al. [22], which estimates the effectiveness of mask wear, reports an OR of 0.85; however, the upper limit of the confidence interval is 1.01. In addition, several of the included trials have few or no events, and sometimes no events in the two arms compared (surgical mask vs. FFP2 mask). Double-zero-event studies can affect the estimation of the effect size [30,31]. Moreover, because MA relies on multiple distributional assumptions, it can be difficult to perform MA with low event rates or few studies [73].

Among the COVID-19 prevention strategies, mask use is the one that has led to the most comparative clinical trials. These are essentially observational studies; the only randomized study currently available is from Loeb et al. [24].

MA does not systematically detail mask wear modalities and comparison strategies. For example, in a healthcare setting, the mask may be worn continuously or not, in the presence or absence of an aerosol-generating procedure. We can also compare mask use with no mask use [34], the N95 mask with the surgical mask (or with certain types of cotton masks, such as the three-layer masks used in some countries).

For some COVID-19 prevention strategies, MA is rare or difficult to perform with the available data. This is the case for eye protection, for example. Byambasuren et al. [74] mention that they were unable to perform a meta-analysis due to the high heterogeneity of the studies included in their literature review. Five non-randomized studies (and therefore subject to numerous biases) were available: three before-and-after studies, one case-control study, and one retrospective cohort. Their data also show that one of the arms in one of the before-after studies had a “zero event”. Moreover, uncontrolled before-after studies are not recommended [75].

Some MA reports on the effectiveness of COVID-19 prevention strategies include studies whose endpoint is respiratory infections, including COVID-19. This is the case in the umbrella MA from Lu et al. [32]. The estimates are therefore biased (indirectness bias).

Thus, the strength of our review is to individually detail the effectiveness of several non-pharmacological preventive measures for COVID-19. Indeed, our review tried to address all types of measures by synthesizing the data from the latest articles published to date in a large and dynamic field of research. Some of the recently published MA articles, by including more articles, allowed a more precise estimation of the effect measure, such as the odds ratio (or relative risk, or hazard ratio, etc.).

Of note, our review only considered issues related to the non-pharmacological prevention of COVID-19. The pharmacological aspects of prevention, vaccination, and prophylactic drugs (such as monoclonal antibodies used to prevent infection and disease progression) have not been addressed here. Despite the publication of numerous MA and LR articles, there are still unanswered questions. Regarding masks in particular, the superiority of N95/FFP2 over surgical masks in the prevention of COVID-19 has not yet been demonstrated. The same applies to the modalities of mask wear in the hospital, continuous or intermittent wear, etc. Fang et al. and the related letters to the editor have discussed that, compared to the surgical mask, the effectiveness of the N95 respirator in the patient care area cannot be definitively determined [76].

Finally, our review provides updated data on several non-pharmacological measures, such as precautions, air and droplet precautions, mask wearing, adequate room ventilation, and air treatment, which are effective in preventing the occurrence of COVID-19.

## Figures and Tables

**Table 1:** Effectiveness of mask, eye protection and other personal protective equipment (Health care workers).

Author	Study Type	Publication Date	Population	Intervention	Comparator	Outcome	Result (95% CI)	Comments
Kunstler et al. [22]	MA 21 studies (only 1 RCT; 13 studies for MA on mask effectiveness)	2022	HCW	N95 mask	Surgical mask	SARS-CoV-2 infection	OR: 0.85 (0.72–1.01)	“High risk of bias” (18/21). Several zero-event studies [30,31]
Loeb et al. [24]	RCT	2022	HCW	N95 mask	Surgical mask	COVID-19 (RT-PCR test)	HR: 1.14 (0.77–1.691)	Potential acquisition of SARS-CoV-2 in the community. Heterogeneity: self-reported adherence; baseline difference between countries: antibodies, circulating variants, vaccination coverage…
Lu et al. [32]	Umbrella MA 10 studies (4 for HCW)	2023	HCW	“N95 or equivalent”	“Medical mask”	“respiratory viral infections (laboratory confirmed) and clinical respiratory illness”	OR: 0.84 (0.73–0.96)	High risk of bias: indirectness [33]
Wu et al. [34]	MA of RCT. 6 studies (4 for medical staff)	2023	HCW	Mask (“N95 or surgical mask”)	No mask	COVID-19	OR: 0.11 (0.01–0.97)	Several zero-event studies [30,31]
Belan et al. [29]	Matched case-control study (nationwide) 2076 cases and 2076 matched controls	2022	HCW	N95 mask	Surgical mask	“Laboratory confirmed cases of COVID-19” (RT-PCR or antigenic test)	aOR: 0.85 (0.55–1.29)	Use of an online questionnaire. Low response rate (cases and controls)
				Eye Protection (goggles or faceshield)	No eye protection		aOR: 0.57 (0.37–0.87)	
				Gown	No Gown		aOR: 0.58 (0.34–0.97)	
				Apron	No Apron		aOR: 1.47 (1.00–2.18)	
				Gloves	No gloves		aOR: 1.44 (0.87–2.39)	
Hajmohammadi et al. [35]	MA of case-control studies 4 studies.	2023	HCW	Mask: “any type of mask”, “medical mask”, or “high performance filtering mask”	Not available. No mask?	“COVID-19 infection”	OR: 0.33 (0.15–0.73)	Risk of bias from the Newcastle-Ottawa Scale [36]: Low risk (between 7 and 9). High heterogeneity (I2 = 82.6%)
				PPE	No PPE	“COVID-19 infection”	OR: 0.41 (0.31–0.54) aOR: 0.35 (0.03–0.67) based on 3 studies	Risk of bias from the Newcastle-Ottawa Scale: low risk (between 7 and 9).

HCW: Health Care Worker; PPE: Personal Protective Equipment. MA: meta-analysis. RCT: Randomized Controlled Trial; MA: Meta-analysis; obs: observational study. OR: Odds Ratio. aOR: adjusted Odds Ratio. RR: Risk Ratio. HR: Hazard Ratio. 95% CI: 95% Confidence Interval.

## Data Availability

Not applicable.
